# Interhemispheric communication during haptic self-perception

**DOI:** 10.1098/rspb.2022.1977

**Published:** 2022-12-07

**Authors:** Gaiqing Kong, Antonio Cataldo, Miruna Nitu, Lucile Dupin, Hiroaki Gomi, Patrick Haggard

**Affiliations:** ^1^ Institute of Cognitive Neuroscience, University College London, Alexandra House, 17–19 Queen Square, London WCIN 3AZ, UK; ^2^ Neuroscience Research Centre of Lyon, INSERM U1028—CNRS UMR5292, Inserm Building, 16 avenue du doyen Lépine, 69500 Bron, France; ^3^ Institute of Philosophy, University of London, Senate House, Malet Street, London WC1E 7HU, UK; ^4^ Institut de Psychiatrie et Neurosciences de Paris, Inserm U 1266—Université de Paris—Hôpital Sainte-Anne, Paris, France; ^5^ NTT Communication Science Laboratories, Nippon Telegraph and Telephone Corporation, Atsugi, Japan; ^6^ Chaire Blaise Pascal de la Région Ile de France, Laboratoire de Neurosciences Cognitives et Computationnelles, Département d'Etudes Cognitives, Ecole Normale Supérieure, PSL University, Paris, France

**Keywords:** haptic perception, self-touch, interhemispheric communication, sensorimotor integration, force–geometry illusion

## Abstract

During the haptic exploration of a planar surface, slight resistances against the hand's movement are illusorily perceived as asperities (bumps) in the surface. If the surface being touched is one's own skin, an actual bump would also produce increased tactile pressure from the moving finger onto the skin. We investigated how kinaesthetic and tactile signals combine to produce haptic perceptions during self-touch. Participants performed two successive movements with the right hand. A haptic force-control robot applied resistances to both movements, and participants judged which movement was felt to contain the larger bump. An additional robot delivered simultaneous but task-irrelevant tactile stroking to the left forearm. These strokes contained either increased or decreased tactile pressure synchronized with the resistance-induced illusory bump encountered by the right hand. We found that the size of bumps perceived by the right hand was enhanced by an increase in left tactile pressure, but also by a decrease. Tactile event detection was thus transferred interhemispherically, but the sign of the tactile information was not respected. Randomizing (rather than blocking) the presentation order of left tactile stimuli abolished these interhemispheric enhancement effects. Thus, interhemispheric transfer during bimanual self-touch requires a stable model of temporally synchronized events, but does not require geometric consistency between hemispheric information, nor between tactile and kinaesthetic representations of a single common object.

## 1. Introduction

Haptic perception involves integrating multiple, qualitatively different sources of information into a coherent percept of the object we are touching [1,2]. For example, when we encounter a bump on a surface during the haptic explanation, the brain combines information about the resistance to the hand's forward movement, and about tactile pressure changes on skin. The integration of tactile and kinaesthetic signals can explain a well-established force–geometry illusion [3]. In this phenomenon, applying forces against or along with the movement of a finger exploring a flat surface produces the illusory perception of bumps or holes in the surface.

The force–geometry illusion and similar haptic phenomena [4] thus reveal tactile–kinaesthetic integration during *unimanual* haptic perception. However, a relatively smaller number of studies have investigated the interhemispheric transfer of tactile and motor information during *bimanual* haptic perception. Dupin and colleagues delivered tactile signals to one hand and synchronous kinaesthetic signals to the other. The tactile sensations in the feeling hand were modulated by the movements of the other hand [5]. Similarly, the direction of either active or passive finger movements of one hand can bias the perceived orientation of tactile stimuli simultaneously delivered to the other hand [6]. These results show an interhemispheric influence of kinaesthetic information over tactile perception. Here, we aim to complement this line of research by investigating the effect of interhemispheric transfer of tactile stimuli on kinaesthetic perception.

Most haptic studies—including those mentioned above—focus on how active touch provides accurate information about external objects. However, the first object of haptic exploration is our own body through self-touch. Self-touch behaviours, such as hand-to-face movements and bimanual manipulation are some of the earliest and most frequent haptic experiences in the life of a human being [7,8]. Arguably, they also constitute the richest haptic experience possible. When the surface that we are haptically exploring is our own skin (as when feeling a pimple, or examining our body for a lump under the skin), besides the tactile and kinaesthetic information from the moving effector, the brain additionally receives tactile sensory information from the touched skin. To the best of our knowledge, a few studies investigated how the brain combines all these different sources [9,10]. Most studies assume that the brain acquires and uses internal models of body-object interactions to combine the weighted tactile and movement information, to estimate properties such as object size [1,11] or compliance [12]. However, the brain might alternatively use a hierarchical approach, prioritizing information from one submodality over other. These alternative hypotheses are difficult to test because of the normal tight coupling between movement and touch during haptic self-exploration.

**Figure 1.**
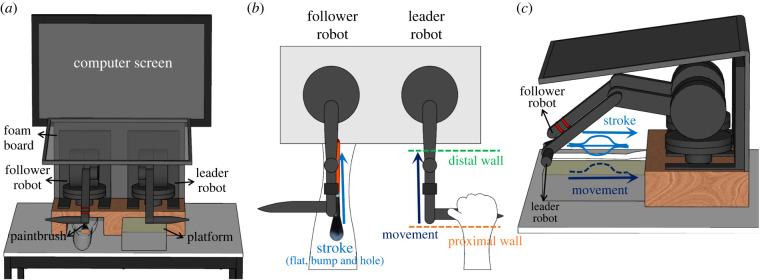
Experimental set-up and stimuli. (*a*) Front view of the leader–follower robotic set-up. (*b*) Top view of the experimental set-up. Participants made two proximo-distal movements with their right hand while holding the leader robot, and simultaneously felt a corresponding stroke on the left forearm from a brush attached to the follower robot. (*c*) Lateral view of the experimental set-up. Resistance was added to both right-hand movements in order to generate two illusory haptic bumps of different sizes. Participants judged which of the two bumps felt larger. The trajectory of the leader robot was replicated by the follower robot, which stroked the participant's left forearm with a paintbrush. The tactile stroking included one of three task-irrelevant modulations of tactile contact force. These achieved the perception of a ‘tactile bump’, a ‘tactile hole’ and a ‘tactile flat’ contact, corresponding to temporarily increased, decreased or unchanged pressure on the left forearm, respectively (electronic supplementary material, videos S1 and S2). (Online version in colour.)

We developed a novel self-touch version of the force–geometry illusion to investigate the interhemispheric communication in bimanual haptic processing (figure 1). This paradigm allowed us to break the congruence between movement and tactile force that characterizes normal self-touch, by decoupling the movement of one hand from its tactile consequences for the other hand. Participants made two successive movements with the right hand while holding a haptic force-control robot. A brief increase in resistance was added to both movements. The resistance elicited the illusion of moving over a bump in the surface [3]. Participants were asked to judge ‘which movement did you feel contained the larger bump?’. The right-hand movements were reproduced by a second, follower robot, which delivered simultaneous stroking touches to the left forearm, as if the participant had been directly stroking their left forearm with their right hand. Although this situation lacks the direct skin-to-skin contact of natural self-touch, tool-mediated self-touch experiences are frequent in daily life, for example, while wearing gloves, or using a hairbrush [13].

The follower robot briefly increased or decreased tactile pressure on the left forearm, in synchrony with the right hand resistance increase. Thus, while the right-hand movement signalled the presence of a haptic bump, the left follower robot delivered tactile signals consistent with the experience of encountering either a bump, or a hole. Crucially, the tactile signals to the left forearm were irrelevant to the task of judging the bump encountered by the right hand. However, tactile information might be automatically integrated interhemispherically, in which case it should alter participants' judgements about the amplitude of the bump felt by the right hand. This paradigm allowed us to investigate what sensory information is shared between hemispheres in bimanual self-touch perception.

## 2. Material and methods

### (a) Participants

A total of 40 participants took part in Experiment 1–2 (Experiment 1: 20, 10 females, mean age ± s.d. = 23.4 ± 4.1; Experiment 2: 20, 11 females, mean age ± s.d. = 24.7 ± 4.6), and six in Control Experiments 3 and 4 (four females, mean age ± s.d. = 23.7 ± 5.1).

All participants were naive to the purpose of the study and reported normal or corrected-to-normal vision, no abnormalities of touch and no neurological history. All participants provided written informed consent before the beginning of the experiment and received a payment for their participation. Procedures were approved by the Research Ethics Committee of University College London and adhered to the ethical standards of the Declaration of Helsinki except for registration in a database. See the electronic supplementary material for the inclusion criteria of each experiment.

### (b) Experimental set-up

Participants sat in front of a computer screen with their left arm on a fixed moulded support (figure 1), and their right arm on an articulated armrest support (Ergorest, series 330011, Finland). Their vision of the hand, arm and the robotic set-up was blocked throughout the experiment by a horizontal foam board placed at the chest level. The sensorimotor self-touch stimulation was implemented using two six-degrees-of-freedom robotic arms (3D Systems, Geomagic Touch X, SC, USA) linked in a computer-controlled leader–follower system. The robotic system was controlled via a customized C++ program (Microsoft Visual Studio C++ Express 2010), which also recorded the kinematic data from both robotic arms. In the leader–follower system, any movement of the right-hand leader robot was reproduced by the follower robot with an estimated lag of 2.5 ms [9]. Two ‘virtual walls’ were created using the force-feedback system of the leader robot to define the starting position and ending position of the right-hand movements (figure 1).

Participants were asked to hold the handle of the leader robot between the thumb and fingers of their right hand and to move from the proximal wall to the distal wall (figure 1). They then lifted the robot in the air to return to the starting position at the proximal wall. A soft flat paintbrush (12.7 mm width) attached to the handle of the follower robot stroked the dorsum of the participant’ left forearm providing a gentle tactile stimulation that was spatially and temporally synchronized with the movements of the leader robot. Thus, moving the leader robot handle back and forth on the platform with the right hand produced a perception of mediated self-touch, like stroking one's own left forearm with a paintbrush.

#### (i) Induction of the haptic bump illusion with the right hand

Virtual bumps [3] were created by using the force feedback of the leader robotic arm. A ‘bump’ zone was created by applying resistance forces opposed to the participants right-hand movement. Resistance was generated with the following cosine function:

2.1 $$Resistance=B_{peak}\times \left\{cos\left[\left(z-\frac{B_{start}+B_{end}}{2}\right)\times \left(\frac{2\pi }{B_{start}-B_{end}}\right)\right]+1\right\},$$

where ‘*B*_peak_’ defines the peak of the bump along the bump zone, while ‘*B*_start_’ and ‘*B*_end_’ represent the starting and ending position of the bump zone (figure 1).

The right-hand movement extent was 80 mm. Resistances generated with function (2.1) were added to decrease movement velocity while moving through the ‘bump’ zone, which began 20 mm from the proximal wall, and extended for 50 mm. The resistance was applied only during proximo-distal movement (the participant raised their right hand during the return movement, and there was no contact of the paintbrush with the left hand). This set-up gave the illusion of encountering a bump-like surface [3] (figure 1).

#### (ii) Tactile stimulation on the left forearm

We manipulated the force feedback of the follower robot to create three different tactile stimulation scenarios on the left forearm, coupled with the haptic bump illusion experienced by the right hand (table 1). In the ‘tactile-bump’ stimulation, the pressure applied to the left forearm by the follower robot *increased* in synchrony with the resistance applied by the leader robot to the participant's movement (see electronic supplementary material, video S1). Conversely, in the ‘tactile-hole’ stimulation, the pressure applied by the follower robot *decreased* (electronic supplementary material, video S2). Finally, in ‘tactile flat’ trials, the paintbrush stroked the participants’ left forearm without any change in pressure. Tactile pressure modulation was achieved by vertically moving the paintbrush 5 mm down or 5 mm up in tactile-bump and tactile-hole trials, respectively (figure 1). The experimenter verified that the brush remained in contact with the skin. For more information on the forces exerted by the follower robot on the left forearm, see the electronic supplemental material.

**Table 1.** Design of Experiment 1 and Experiment 2.

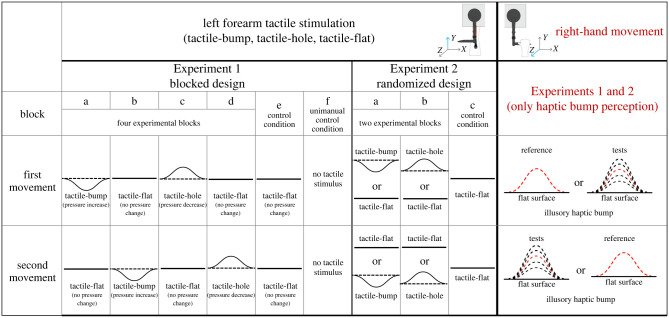

### (c) Experimental design

#### (i) Experiment 1—blocked design

We adopted the method of constant stimuli, presenting a reference and a test illusory bump of different sizes in each trial and in unpredictable order. The reference bump size was always 3 N indicated by the peak of the added resistance to the right hand, while the test stimulus was randomly selected from five possible sizes (±1 N, ±0.5 N and 0 N relative to the reference bump). There were 15 repetitions for the ±1 N test movement, 20 repetitions for the ±0.5 N test movement and 30 repetitions for the 0 N test movement (i.e. equal to reference), giving100 trials for each block.

The type of tactile stimulation and its presentation order (i.e. during the first or second movement) were both blocked (table 1). In each block, one of three possible task-irrelevant tactile stimulations (tactile-bump, tactile-hole or tactile-flat) was predictably delivered to the participant's left forearm, in either the first or the second movement. Participants performed six different blocks, corresponding to each of the different combinations of the three tactile stimulations and the two presentation orders (table 1; see electronic supplementary material for a more detailed description of each block). For the four experimental blocks (a)–(d), the presentation order of the tactile stimuli in the two movements of each trial was held constant. For example, in block (a), the tactile-bump was always presented in the first right-hand movement, and the tactile-flat was always presented in the second right-hand movement. For the control block (e, *baseline: no tactile pressure change*), given that randomizing the presentation order of the two flat stimuli would simply generate a duplicate of the same block, instead we performed block (f) as a further unimanual control block where no tactile stimuli were delivered on the left forearm at all.

The order of the six blocks was counterbalanced across participants. The four experimental blocks led to a 2 (tactile feature: bump, hole) × 2 (movement: Test, Reference) within-subject design. Each block lasted about 15 min and the whole experiment lasted about 2 h.

#### (ii) Experiment 2—randomized design

In Experiment 2, we randomized the presentation order of the tactile stimulation in the two movements in each trial, producing two experimental blocks (a) and (b) and one control block (c), table 1 and electronic supplemental material. The order of the three blocks was counterbalanced across participants. Thus, in Experiment 2, the tactile stimulation had no predictable relation with the right haptic bump, other than temporal synchrony. For example, in block (a), the tactile stimulation presented in the first movement could be a tactile-bump or a tactile-flat. The control block (c) served as a control condition (*baseline: no tactile pressure change*) and was similar to block (e) in Experiment 1. As the two control conditions in Experiment 1 produced highly similar results (see electronic supplemental material), we did not include a unimanual control condition (no tactile stimulus) in Experiment 2.

Similar to Experiment 1, the data were analysed using a 2 (tactile-bump, tactile-hole) × 2 (test movement, reference movement) within-subject design. The two experimental blocks (a) and (b) included 192 trials (16 repetitions for the ±1 N test movement, 20 repetitions for the ±0.5 N test movement and 24 repetitions for the ± 0 test movement). The control block (c), instead, included 96 trials (8 repetitions for the ±1 N test movement, 10 repetitions for the ±0.5 N test movement and 12 trials for the ± 0 test movement). Each block lasted about 35 min and the whole experiment lasted about 2 h.

Note that in both Experiment 1 and Experiment 2 the tactile stimulation was always applied to the left forearm, while the haptic-bump perception was always tested on the right hand. Moreover, the tactile-bump or tactile-hole were both randomly associated with the haptic-bump reference or test movement. The identity of the tactile feature (bump or hole) was predictable in both experiments. Whether this tactile feature would occur in the first or second movement was predictable in Experiment 1, but unpredictable in Experiment 2.

#### (iii) Control Experiments 3 and 4

In two control experiments, we tested whether increases and decreases in contact force during stroking of the left forearm were indeed perceptually discriminable. We reasoned that discriminability would be the first prerequisite for qualifying the two tactile stimuli as tactile bumps and holes, respectively.

Accordingly, in Experiment 3 participants were required to make a same-different judgement about two left-forearm tactile strokes presented in quick succession. There were four possible combinations (bump–bump, hole–hole, bump–hole, hole–bump) and each combination was presented in a randomized order. Each of the four combinations was repeated ten times, resulting in a total of 40 trials. Given that discriminability is necessary but not sufficient to characterize the tactile stimuli as being congruent with a bump or a hole, in Experiment 4 we delivered ten trials of increased pressure (tactile-bump) and ten trials of decreased pressure (tactile-hole) on the participant's left forearm in a randomized order. Participants were asked to report whether they felt and increase or decrease in the pressure on their left arm.

In both control experiments, the experimenter moved the leader robot to produce the tactile force on the participant's left forearm via the follower robot. Thus, the participants just passively received the tactile sensation on their left forearm, while resting their right hand in their lap throughout the control experiments. Experiment 4 was performed directly after Experiment 3.

### (d) Procedure

In each experiment, each trial started with a black screen and a white central fixation-cross, prompting the participant to move the handle of the leader robot to the start position at the proximal wall. Then, the fixation-cross disappeared and a beep prompted the participant to perform a proximo-distal movement. Another beep was also used to signal the participant once they arrived at the distal wall. The text ‘First’ was presented on the screen once the fixation-cross disappeared, when there are two consecutive movements in a trial, to indicate the start of the first movement. Then it was replaced by the text ‘Second’ once the first movement ended and the handle was brought back to the starting position to indicate the start of the second movement. Based on the purpose of each experiment, the question (e.g. ‘which bump was larger, first or second?’) was presented on the screen at the end of each trial.

Participants responded verbally and the experimenter entered their response via a keyboard. Participants then clicked a button on the handle of the leader robot to indicate response completion and bring the handle back to the starting position for the next trial. Participants had a break after each session in each experiment and were allowed to rest between trials. The participants were warned by the experimenter when they moved too fast or too slowly (movement time, less than 1.5 or greater than 2.5 s). Before testing, the participants were familiarized with the movements and the questions.

### (e) Statistical analyses

For the two main experiments, the percentage of participants’ responses that judged the bump in the test movement as being larger than the reference bump was calculated individually for each participant. The five data points (±1 N, ±0.5 N and 0 N relative to the reference) were fitted onto a psychometric curve using the logistic function [14] with the Palamedes Toolbox [15]. For each participant, the transitional threshold, that is, the point of subjective equality (PSE) at which the test bump is judged equal to the reference bump, was calculated by estimating 50% of the reporting on the fitted curve. The just noticeable difference (JND), an indicator of the sensitivity of bump size discrimination, was calculated as half of the interquartile range of the psychometric curve. We calculated the PSEs and JNDs when a tactile-bump or tactile-hole was added to a reference or test movement respectively, generating four experimental conditions: *tactile bump* *+* *ref* (a tactile-bump was added to a reference movement), *tactile bump* *+* *test* (a tactile-bump was added to a test movement), *tactile hole* *+* *ref* (a tactile-hole was added to a reference movement) and *tactile hole* *+* *test* (a tactile-hole was added to a test movement) conditions in both experiments. We also calculated the PSEs and JNDs for the two control conditions (*baseline: no tactile pressure change* and *unimanual control*: *no tactile stimulus*) in Experiment 1 and one control condition (*baseline: no tactile pressure change*) in Experiment 2. Please see the detailed statistical analyses in the electronic supplemental material.

## 3. Results

### (a) Experiment 1—blocked design

This experiment used a blocked design to investigate the contribution of tactile stimuli delivered to the left forearm to the perception of an illusory bump haptically explored with the right hand.

**Figure 2.**
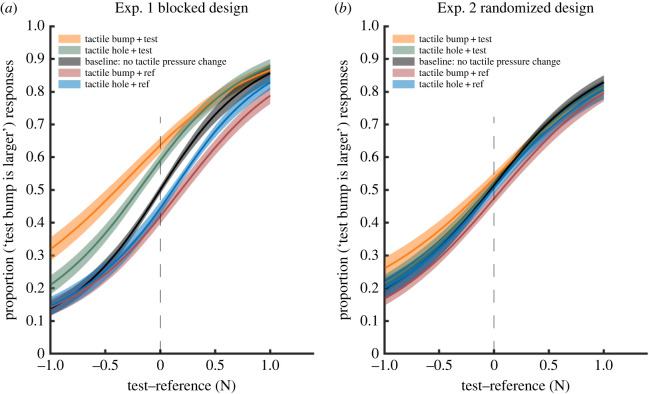
Average psychometric curves for (*a*) Experiment 1, blocked design and (*b*) Experiment 2, randomized design. The ‘proportion of the test stimulus was larger’ was fitted as a function of the difference between test and reference (test–reference). Grey curve is the control condition that served as a baseline (no tactile pressure change), while coloured curves indicate experimental conditions, with different tactile events contributing to the haptic percept. Shaded area indicates the standard error of the mean. Vertical dashed line indicates the bump size in the reference movement. The reference bump size was always 3 N indicated by the peak of the added resistance, and the test stimulus was randomly selected from five possible sizes (±1 N, ±0.5 N and ±0 N relative to the reference bump). See Methods for details. (Online version in colour.)

Figure 2 shows the average psychometric curves for the four experimental conditions and the baseline condition, see the individual psychometric functions from each participant in electronic supplementary material, figures S8 and S9. As the two control conditions produced highly similar results (see electronic supplemental material), the unimanual control condition is omitted from figure 2. The sigmoid shape shows that resistance to the right hand's movement successfully induced a bump-like haptic illusion, with stronger resistive forces to the right hand producing the percept of a larger bump. Further, task-irrelevant pressure changes to the left forearm influenced the perceived amplitude of these bumps, shifting the participants’ PSE. A leftward shift means that simultaneous tactile stimulation to the left arm biased the perception of the bump evoked by resistance to the right hand during the test movement, making the test bump feel larger. A rightward shift means that tactile stimulation made the reference bump feel larger.

To investigate how tactile and movement information are combined in haptic perception, we ran a 2 × 2 ANOVA with factors of tactile feature (tactile-bump, tactile-hole) and whether the feature was presented during the test or the reference movement. The tactile feature itself (bump or hole) was not significant (*F*_1,19_ = 3.43, *p* = 0.08, $\eta_{p}^{2}\;=0\;.15$). The main effect of test/reference was highly significant, indicating that adding either tactile-bump features or tactile-hole features made the right-hand test stimulus feel ‘bumpier’ (*F*_1,19_ = 24.08, *p* < 0.001, $\eta_{p}^{2}\;=0\;.56$). The interaction of these factors showed that a tactile hole produced the same direction of effect as a tactile bump, though to a lesser extent (interaction effect (*F*_1,19_ = 5.08, *p* = 0.036, $\eta_{p}^{2}\;=0\;.21$). Thus, any tactile pressure changes to the left hand, whether increases suggestive of a bump or decreases suggestive of a hole, led to the right-hand haptic percept feeling bumpier. The *sign* or congruence of tactile pressure change was irrelevant to the right-hand percept. ANOVA on JNDs showed no significant effects (table 2).

**Table 2.** The average PSEs and JNDs for five conditions in Experiment 1 and Experiment 2.

	Exp. 1		Exp. 2	
	PSEs (N) mean (s.e.)	JNDs (N) mean (s.e.)	PSEs (N) mean (s.e.)	JNDs (N) mean (s.e.)
*tactile bump + test*	2.47 (0.11)	1.48 (0.16)	2.81 (0.07)	1.11 (0.15)
*tactile hole + test*	2.76 (0.05)	1.92 (0.20)	2.92 (0.06)	0.88 (0.10)
*baseline: no tactile pressure change*	3.01 (0.03)	2.11 (0.22)	2.92 (0.03)	0.85 (0.11)
*tactile bump + ref*	3.20 (0.09)	1.83 (0.21)	3.09 (0.05)	0.89 (0.15)
*tactile hole + ref*	3.06 (0.06)	1.99 (0.23)	2.94 (0.07)	1.05 (0.22)
*unimanual control: no tactile stimulus*	2.96 (0.03)	2.12 (0.20)		

Two unisensory control Experiments 3 and 4 (see detailed results in the electronic supplemental material) confirmed that participants did indeed perceive the differences between pressure increases versus decreases on the left forearm. Thus, specific information about *which* tactile event had occurred, bump or hole, was perceptually available, but yet was not integrated with the right-hand haptic percept in Experiment 1. That is, the *occurrence* of tactile events was transferred interhemispherically for integration with right-hand movement information, but the *sign* of these events, pressure increases versus decreases, was not transferred. Further, informal debriefing after Experiment 1 confirmed that participants discriminated between the tactile events on the left forearm: participants were invited to draw the profile of the pressure changes they felt on their left forearm. Their drawings showed that they correctly distinguished pressure increases from decreases (an illustrative set of drawings is shown in electronic supplementary material, figure S4). Thus, loss of tactile sign information from the left hand did not simply reflect participants’ imperceptions, or selective attention to the right, moving hand, but instead reflected a limitation on how tactile and movement information were interhemispherically integrated.

### (b) Experiment 2—randomized design

In Experiment 2, we randomized the presentation order of tactile stimulation, so participants could not predict whether the tactile event would occur during the first or the second movement. Randomized designs mean that participants cannot form a precise prior, even for a single stimulus like the left tactile event [16]. The sign of the tactile event (bump or hole) remained a blocked factor, and was, therefore, predictable: only the time of event occurrence was unpredictable (see Methods).

**Figure 3.**
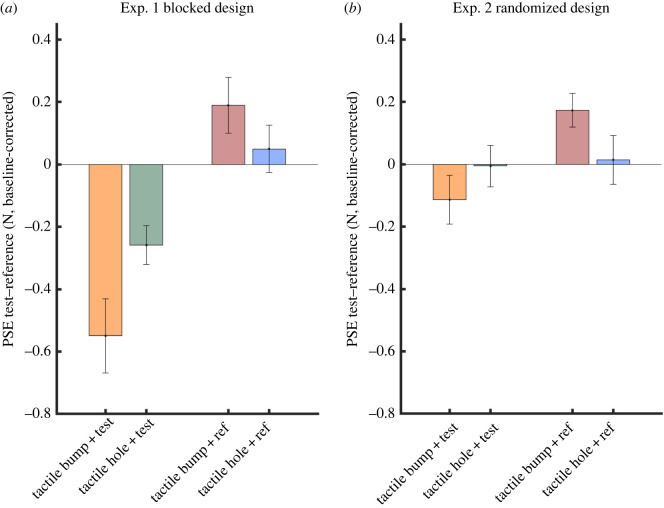
PSEs of Experiment 1 (blocked design, (*a*)) and Experiment 2 (randomized design, (*b*)). Bars of different colours denote the four experimental conditions: *tactile bump + test*, *tactile hole + test, tactile bump + ref* and *tactile hole + ref*, respectively. The error bar indicates the standard error of the mean across participants. The size of the reference bump was always 3 N, and the test stimulus was randomly selected from five possible sizes (±1N, ±0.5 N and ±0 N relative to the reference bump). We calculated the difference in PSE for trials where tactile information was added to the test bump versus the reference bump, and then corrected this value by subtracting each participant's PSE in the *baseline: no tactile pressure change* condition. (Online version in colour.)

Twenty participants took part in Experiment 2 (11 females, mean age ± s.d. = 24.7 ± 4.6). A two-way repeated measures ANOVA on PSEs again showed no main effect of bumps versus holes (*F*_1,19_ = 0.38, *p* = 0.54, $\eta_{p}^{2}\;=0\;.02$), a significant main effect of whether tactile events were added to the test or the reference (*F*_1,19_ = 8.08, *p* = 0.01, $\eta_{p}^{2}\;=0\;.30$), but no interaction (*F*_1,19_ = 1.94, *p* = 0.18, $\eta_{p}^{2}\;=0\;.09$). Importantly, the effect of test versus reference was markedly smaller in Experiment 2 than in Experiment 1. This was confirmed in an across-experiment comparison, which showed a highly significant interaction between the factors of Experiment (1, blocked versus 2, randomized) and test–reference (*F*_1,38_ = 9.60, *p* = 0.004, $\eta_{p}^{2}\;=0\;.20$). *Post hoc* simple effects analysis showed that this interaction arose because right bump perception was strongly influenced by the left tactile events when these were blocked (Exp. 1, test versus ref: *F*_1,38_ = 38.38, *p* < 0.001, $\eta_{p}^{2}\;=0\;.50$), but was less, and non-significantly influenced by left events when these were randomized (Exp. 2, test versus ref: *F*_1,38_ = 3.28, *p* = 0.08, $\eta_{p}^{2}\;=0\;.08$). Expressing the interaction effect of figure 3 as a single quantity showed that randomizing the occurrence of the tactile pressure change reduced the size of the interaction effect in Experiment 2 to 61.9% of that in Experiment 1. ANOVA on JNDs again showed no significant effects (table 2).

Finally, we compared the movement trajectory, velocity, and acceleration of Experiments 1 and 2, and the unimanual control experiments, and found very similar patterns in all cases (electronic supplementary material, figures S5–S7).

These results indicate that intermanual transfer was strongly reduced when the left tactile events were randomized compared to when the left tactile events were blocked. This rules out the possibility that touch–movement interaction simply reflects generalized alerting due to additional stimulation. Effects of alerting should be, if anything, stronger in a randomized than in a blocked design, yet our interference effects were significantly weaker. Rather the interhemispheric modulation requires that participants predict the occurrence of tactile event on the left forearm, and then combine it with movement information from the right hand.

## 4. Discussion

This study aimed to investigate interhemispherical transfer of tactile and kinaesthetic information during self-touch. Using a novel self-touch version of the force–geometry illusion [3] we decoupled the sensorimotor signals involved in haptic self-exploration by delivering task-irrelevant tactile information that were either congruent or incongruent with the kinaesthetic illusion of encountering a bump.

Our first main finding is that haptic perception during self-touch involves the transfer of information about the *occurrence* of tactile events, but not detailed information about their *content*. Our experimental design specifically aimed at contrasting unsigned, occurrence-based integration of events, and signed, geometry-based integration of object information, and so was well able to address this research question. To be specific, tactile force profiles consistent with either positive asperity (bumps) or negative asperity (holes) both produced comparable shifts in perceptual judgements made with the right hand—and these shifts always had effects in the same direction: namely, making bumps encountered by the right hand feel larger. Second, we found that this intercommunication required *predictable* co-occurrence of a tactile and a motor event, since effects were present in blocked Experiment 1, but not in randomized Experiment 2.

Taken together, these findings suggest that participants do not learn an intermanual model of a geometric object corresponding to the asperity. Instead, they learn a consistent *temporal* relationship between tactile and kinaesthetic events, without integrating these two events into a single *geometric* percept. That is, sensorimotor integration in self-touch appears to be oriented towards perceiving temporal *events*, rather than perceiving spatial, geometric *objects.* In this respect, our study differs from previous haptic studies, which typically focused on the perception of object geometry [1,2]. In our study, the tactile event could differ from the right hand's proprioception in the sign of the implied asperity, which is crucial for geometry. However, the two signals were consistent in other aspects, notably in the synchrony of their onsets. Future research could potentially explore in what respects left and right-hand stimuli must be similar for interhemispheric transfer to occur. Causal inference models [17] would suggest that a highly dissimilar left tactile stimulus (e.g. a vibration burst) might not contribute to asperity perception.

In other multisensory paradigms, visual and auditory motion are not integrated as directional signals carrying information about a common object, but as higher-level ‘decision signals’ [18–20]. Gori and colleagues found that visual and tactile motion integration could take two forms: vectorial integration of (signed) information at a low-level of direction-specific sensory signals, and probabilistic (unsigned) facilitation at a higher decision-level stage [2,21]. Our tactile–kinaesthetic intermanual integration resembles the latter process.

Simultaneous somatosensory electrical [22], thermal [23] and spatial [24] stimuli presented to two hands can be combined to produce summed or averaged estimates. However, those studies always involved the perceptual integration of two stimuli in the *same modality*. By contrast, our study investigated the intermanual integration of two separate submodalities of somatosensory perception, namely cutaneous touch and kinaesthetic information. Neural, computational and behavioural studies [25–27] demonstrate the importance of integrating tactile and kinaesthetic information for functional somatosensation [3,5]. However, most previous studies described this integration, without probing it experimentally, perhaps because of the difficulty of decoupling movements from their tactile consequences.

Our set-up involves a tool-mediated version of self-touch. Everyday self-touch involves a distinctive skin-to-skin sensory stimulation of the skin of the touching hand, which our set-up could not emulate. However, the leader–follower robot set-up allowed our experiment to replace the normal tight coupling between haptic and tactile signals, with precise experimental control over the crucial relation between movement and touch. This in turn allowed us to test how the brain combines tactile and movement information in order to form haptic percepts during self-touch. This investigation, therefore, involves a technologically mediated equivalent of self-touch, which contrasts with traditional haptic tasks involving the exploration of external objects [1,11]. It also allows us to distribute tactile and motion information across two different hemispheres. This introduces an element of interhemispheric intercommunication between tactile and movement signals that is common in self-touch, but remains unusual in laboratory haptics studies.

Hayward and colleagues [3,5] showed that kinaesthetic inputs could produce bump perception more readily than hole perception. We accordingly focused here on haptic-bump perception with the right hand during a self-touch situation. In this situation, we found that the time of occurrence of tactile stimulation of the left forearm by the moving right hand, is transferred interhemispherically, whereas information about the sign of tactile force changes, and consequent geometric properties of the implied tactile object, is not. In other words, the final sign of the overall percept was determined by the movement information from the right hand alone, regardless of the sign of the tactile signals from the left arm. This finding suggests a potential dominance of movement information over tactile signals in haptic perception.

Previous studies [3] showed that movement information alone was sufficient for perceiving a bump in a haptically explored surface. On that view, movement information should be sufficient for haptic geometry perception. Our results extend but qualify this movement-dominant view, by showing that tactile sensations on the left forearm associated with bumps and holes during self-touch could enhance the haptic illusion of a bump encountered while moving the right hand, but could never reduce it, relative to a condition with no relevant tactile input to the left forearm. Therefore, even though movement information may be sufficient for haptic perception, tactile information is also integrated. The sign of tactile information seemed to be lost in the integration with movement information, and only unsigned tactile signals were available for integration with movement. Similar patterns of unsigned integration have been reported in other sensory systems. Thus, in vision, unsigned auditory motion information is integrated with position signals to produce a percept of motion, leading to striking illusions [18,20].

Our finding of unsigned, as opposed to signed, integration does not simply reflect a general inability to combine spatial information in self-touch. Indeed, in a recent self-touch experiment [10], we showed that spatial *extent* perception involved *signed* interhemispheric integration, so that tactile strokes that were longer or shorter than a self-touch movement produced positive and negative biases, respectively, in judgements of movement extent. By contrast, in the present experiment, tactile pressure information exhibited *unsigned* interhemispheric integration. We suggest that signed interhemispheric integration may be possible only when two hemispheric signals share a common dimension or common metric format, such as spatial extent. By contrast, tactile–kinaesthetic integration in haptic perception provides a canonical example of two signals that have very different signal formats (pressure changes normal to a surface and velocity changes parallel to a surface, respectively), even though they are potentially informative about a single distal object. We suggest that causal inference involving such different dimensions and signal formats may be based on unsigned integration [17,20], while the integration of signals in the same format may be based on signed information.

Thus, a crucial distinction arises according to how much signal transformation is required before signals are integrated. When signal transformation is not required, or is minimal, signed integration may occur. Conversely, when signals must be transformed into a common format prior to integration, as in the present case of combining movement and tactile cues about asperity, unsigned integration may be found. Traditionally, since Euclid, the geometric perspective has emphasized amodal spatial properties that are independent of presentations in any individual sense modality [28]. Our experimental results suggest that amodal representation, based on transformation and then integration of different sensory signals, is possible, but is, in fact, time-based and event-based, rather than spatial. By contrast to the abstract and static Euclidean geometric perspective, the brain must generate spatial perceptions from time-specific and input-modality-specific signals. This difference may explain the priority of temporal information over the geometric perspective in our data.

Comparing Experiments 1 and 2, we found that tactile information was more strongly integrated with movement information when tactile event occurrence was temporally predictable. The combination of touch and movement information in haptic perception may involve event-based models of temporal synchrony, similar to audio-visual and other multisensory interactions.

Finally, our study approximates haptic exploration of one's own body through self-touch. Self-touch has long been considered a foundational mechanism for spatial perception, and indeed for wider cognition [9,10,29]. Many accounts of sense of self emphasize spatial coherence of multiple bodily signals [30,31], linked to an internal metric model of the body. Our study suggests that sense of self additionally depends on *temporal* predictability, through models that predict synchronized events across tactile and kinaesthetic sensory channels.

## Data Availability

Data and analysis scripts associated with this manuscript can be found on the Open Science Framework repository: https://osf.io/e7qw9/. The data are provided in the electronic supplementary material [32].
